# Autochthonous Transmission of East/Central/South African Genotype Chikungunya Virus, Brazil

**DOI:** 10.3201/eid2310.161855

**Published:** 2017-10

**Authors:** Marcela S. Cunha, Nádia V.G. Cruz, Laila C. Schnellrath, Maria Luiza Gomes Medaglia, Michele E. Casotto, Rodolpho M. Albano, Luciana J. Costa, Clarissa R. Damaso

**Affiliations:** Universidade Federal do Rio de Janeiro, Rio de Janeiro, Brazil (M.S. Cunha, N.V.G. Cruz, L.C. Schnellrath, M.L.G. Medaglia, L.J. Costa, C.R. Damaso);; Instituto de Biologia do Exército, Rio de Janeiro (N.V.G. Cruz, M.E. Casotto);; Universidade do Estado do Rio de Janeiro, Rio de Janeiro (R.M. Albano)

**Keywords:** Chikungunya virus, vector-borne infections, arboviruses, Zika virus, dengue virus, ECSA genotype, viruses, Brazil, mosquitoes, alphaviruses, East/Central/South African Genotype

## Abstract

We isolated East/Central/South African genotype chikungunya virus during the 2016 epidemic in Rio de Janeiro, Brazil. Genome sequencing revealed unique mutations in the nonstructural protein 4 (NSP4-A481D) and envelope protein 1 (E1-K211T). Moreover, all Brazil East/Central/South isolates shared the exclusive mutations E1-M407L and E2-A103T.

Chikungunya virus (CHIKV) is an alphavirus (family *Togaviridae*) transmitted by *Aedes aegypti* and *Ae. albopictus* mosquitoes. Chikungunya fever is characterized by fever, intense polyarthralgia, headache, joint swelling, and rash. Polyarthralgia can persist for several months after the acute phase (*1*). The 3 main CHIKV genotypes are Asian, West African, and East/Central/South African (ECSA), in addition to the ECSA-derived Indian Ocean lineage (*1*). Genetic analysis of CHIKV genomes has shown substitutions in envelope (E) 1 and E2 proteins that affect virus adaptability to *Aedes* mosquitoes. For example, E1-K211E and E2-V264A mutations have been reported to increase CHIKV fitness in *Ae. aegypti* (*2*), whereas E1-A226V and E2-L210Q mutations have been associated with improved adaptability to *Ae. albopictus* mosquitoes (*3**,**4*). The E1-T98A mutation enhances the vector-adaptability effect of E1-A226V, which is otherwise restricted by epistatic interactions between E1-98T and E1-A226V (*4*).

Autochthonous transmission of CHIKV (Asian genotype) in Brazil first occurred in 2014 in Oiapoque, Brazilian Amazon, 1 year after CHIKV introduction in the Caribbean (*1*). Since late 2014, autochthonous cases of the ECSA genotype have been reported in northeastern Brazil (*1*), a region of sustained cocirculation of dengue virus (DENV) for decades and the epicenter of recent Zika virus outbreaks (*5*).

Until late 2015, only a few imported cases of CHIKV (Asian genotype) had been reported in Rio de Janeiro (*6*). However, in December 2015, ten autochthonous CHIKV transmissions were reported, followed by an increase to 11,602 by August 2016, which accounted for 88.9% of reported cases in the state. Of these, 1,868 have been laboratory confirmed as CHIKV, leading to the highest incidence of CHIKV infection in southeastern Brazil (*7*). Nevertheless, the CHIKV genotype associated with the epidemic in Rio de Janeiro remains unknown.

On March 16, 2016, the emergency laboratory of the Brazilian Army Institute of Biology at Rio de Janeiro collected blood samples from a 16-year-old girl and a 29-year-old man who sought care at the associated military hospital. The patients had fever (40°C) for 24–48 h, debilitating polyarthralgia, and myalgia. The man also had exanthema and itching. The diagnostic hypotheses were Zika, dengue, or chikungunya, given their similar clinical symptoms (*5*). Laboratory findings were unremarkable except for leukopenia (2,630 cells/mm^3^ [reference range 4,500‒10,000 cells/mm^3^]) in the man. The patients were not related, lived 13 km apart, and had no history of travel outside the region during the previous 30 days. Both reported persistent mild arthralgia since October 2016. They were informed about this study and provided oral consent.

Blood samples tested negative for CHIKV, DENV, and Zika virus by TaqMan-based real-time reverse transcription PCR (*8*). Nevertheless, virus isolation from blood-cell fraction was successful in Vero cells infected for 48 h (isolate RJ-IB1 from the girl and RJ-IB5 from the man). The isolates had similar growth kinetics in cell culture, reaching highest yields at 48 h after infection (Figure). Virus RNA isolated from cell supernatants tested positive for CHIKV by real-time reverse transcription PCR and negative for DENV and Zika virus (*8*).

**Figure:**
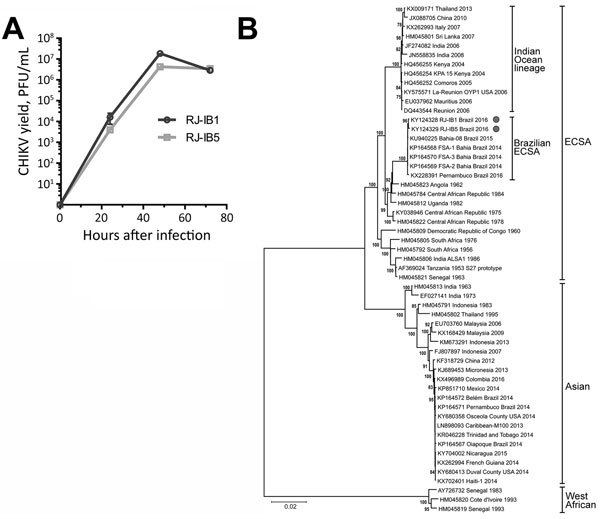
Growth curve and phylogenetic analysis of RJ-IB1 and RJ-IB5 CHIKV isolates, Rio Janeiro, Brazil, 2016. A) Vero cell–amplified RJ-IB1 or RJ-IB5 was titrated and used to infect Vero cells in duplicates at a multiplicity of infection of 0.05. The resulting supernatants were harvested at 0, 24, 48, and 72 h after infection. The production of infectious progeny was determined by plaque assay in Vero cells. B) The full-genome sequences of RJ-IB1 and RJ-IB5 isolates and 53 CHIKV strains representing all known genotypes were aligned with MAFFT (http://mafft.cbrc.jp/alignment/software). Phylogeny inference was performed with MEGA 6 (http://www.megasoftware.net) opting for the neighbor-joining method and kimura-2p model of substitution. Numbers on branches indicate the percentage of bootstrap support from 1,000 replicates. Values >70% are shown. Similar tree topology was obtained with maximum-likelihood method opting for Tamura-Nei model of substitution. Isolates are identified by GenBank accession number, location, and year of CHIKV isolation; genotypes are indicated at right. CHIKV, chikungunya virus; ESCA, East/Central/South African genotype. Scale bar indicates nucleotide substitutions per site.

We used RNA isolated from cell supernatants to sequence the RJ-IB1 and RJ-IB5 genomes. After cDNA synthesis, we amplified 4 overlapping fragments by PCR and sequenced them on an Illumina MiSeq platform after tagmentation-based library construction with the Nextera-XT DNA Sample Prep kit (Illumina, San Diego, CA, USA). We used Geneious (http://www.geneious.com) for de novo genome assembly.

RJ-IB1 and RJ-IB5 mapped within the ECSA genotype with other isolates from northeastern Brazil (Figure). We found no evidence of intergenotypic recombination despite cocirculation of ECSA and Asian strains in Brazil since 2014. The coding region of the consensus genome sequences revealed mutations present in 98.3%–100.0% of the roads mapping to each mutated residue. We detected unique mutations in RJ-IB1 and RJ-IB5 that are absent in other CHIKV isolates: NSP4-A481D in the viral RNA polymerase and E1-K211T in an E1 polymorphic site. A K211E mutation in this same E1 site showed increased CHIKV adaptability to *Ae. aegypti* mosquitoes in a E1-226A background (*2*). Nonetheless, the effects of the E1-K211T substitution in Rio de Janeiro isolates are unknown. Furthermore, we detected unique substitutions in RJ-IB1 (E1-N335D) and RJ-IB5 (NSP2-L27I). Both Rio de Janeiro isolates and the ECSA isolate Bahia-08 share a NSP2-P352A substitution, which is not detected in other Brazil ECSA isolates. In contrast, the Brazil ECSA subgroup shares 2 exclusive mutations (E1-M407L and E2-A103T) that are absent in other ECSA strains. RJ-IB1 and RJ-IB5 genomes do not have the vector-adaptive E1-A226V or E2-L210Q substitutions but do have the E1-T98A substitution also present in other ECSA isolates. We also detected minority variants within the viral population, particularly in RJ-IB1, which showed higher genetic diversity than RJ-IB5 (online Technical Appendix Table, https://wwwnc.cdc.gov/EID/article/23/10/16-1855-Techapp1.pdf). Further investigation of plasma-isolated viruses is necessary to confirm the diversity of minority variants (*9*).

Genetic surveillance and screening for mutations that might alter CHIKV fitness in vertebrates or *Aedes* mosquitoes are crucial. Brazil reports wide geographic distribution of *Ae. aegypti* mosquitoes, with substantial concentrations in northern regions. Conversely, *Ae. albopictus* mosquitoes are concentrated in the subtropical southeastern states (*10*), which can lead to selection of different adaptive mutations in circulating CHIKV strains.

Technical Appendix Minority-variant frequencies within chikungunya virus RJ-IB1 and RJ-IB5 population
