# Supplementary material for: Autochthonous Transmission of East/Central/South African Genotype Chikungunya Virus, Brazil
Source: Emerg Infect Dis. 2017 Oct;23(10):1737–9. doi: 10.3201/eid2310.161855 (PMC5621531; doi:10.3201/eid2310.161855)
Supplement: Technical Appendix — Minority-variant frequencies within chikungunya virus RJ-IB1 and RJ-IB5 population [file 16-1855-Techapp-s1.pdf]

Article DOI: https://doi.org/10.3201/eid2310.161855

Autochthonous Transmission of East/Central/South African Genotype Chikungunya Virus, Brazil

Technical Appendix

Technical Appendix Table. Minority-variant frequencies within chikungunya virus RJ-IB1 and RJ-IB5 population, Rio de Janeiro, Brazil, 2016*

					Variant frequency, %‡	
Genome segment	Nucleotide position	Nucleotide change	Amino acid change†	Protein effect	RJ-IB1	RJ-IB5
NSP1	94	C >T		None	8.5	
NSP1	96	T >A	I8K	Substitution	8.6	
NSP1	585	G >A	R171Q	Substitution	2.2	
NSP1	1,322	A >C		None		11.3
NSP1	1,478	A >G	K469E	Substitution	4.8	
NSP2	2,021	A >C	T115P	Substitution	9.9	
NSP2	3,910	G >A		None	5.0	
NSP3	4,168	T >G		None	9.2	
NSP3	5,338	A >C		None	6.7	
NSP4	5,855	A >C	T26P	Substitution	13.9	
NSP4	5,783	A >T		None		4.9
NSP4	5,862	C >T		Truncation	18.6	8.6
NSP4	6,242	A >C	T194P	Substitution	7.6	
NSP4	6,412	T >C		None	3.8	
NSP4	6,580	A >G		None	4.4	
NSP4	7,187	T >G	F509V	Substitution	8.7	
E3	8,364	C >T		None	2.5	
E2	9,305	A >C	H256P	Substitution	10.2	
E2	9,570	A >G		None	2.3	
E2	9,573	C >G	N345K	Substitution	2.8	
E1	10,291	A >C	T101P	Substitution	16.1	
E1	10,648	A >C		None		5.1

*Base coverage >100× and frequencies >0.5% were analyzed.
†Blank cells indicate synonymous substitutions.
‡Blank cells indicate that the specific variant does not occur in the referred chikungunya virus isolate.
